# Unraveling the intricate relationship between lipid metabolism and oncogenic signaling pathways

**DOI:** 10.3389/fcell.2024.1399065

**Published:** 2024-06-12

**Authors:** Fahad Khan, Deena Elsori, Meenakshi Verma, Shivam Pandey, Safia Obaidur Rab, Samra Siddiqui, Nadiyah M. Alabdallah, Mohd Saeed, Pratibha Pandey

**Affiliations:** ^1^ Center for Global Health Research, Saveetha Medical College and Hospital, Saveetha Institute of Medical and Technical Sciences, Chennai, Tamil Nadu, India; ^2^ Faculty of Resilience, Rabdan Academy, Abu Dhabi, United Arab Emirates; ^3^ University Centre for Research and Development, Chandigarh University, Mohali, Punjab, India; ^4^ School of Applied and Life Sciences, Uttaranchal University, Dehradun, Uttarakhand, India; ^5^ Department of Clinical Laboratory Sciences, College of Applied Medical Science, King Khalid University, Abha, Saudi Arabia; ^6^ Department of Health Service Management, College of Public Health and Health Informatics, University of Hail, Haʼil, Saudi Arabia; ^7^ Department of Biology, College of Science, Imam Abdulrahman Bin Faisal University, Dammam, Saudi Arabia; ^8^ Basic and Applied Scientific Research Centre, Imam Abdulrahman Bin Faisal University, Dammam, Saudi Arabia; ^9^ Department of Biology, College of Science, University of Hail, Haʼil, Saudi Arabia; ^10^ Chitkara Centre for Research and Development, Chitkara University, Himachal Pradesh, India; ^11^ Centre of Research Impact and Outcome, Chitkara University, Rajpura, Punjab, India

**Keywords:** cancer, lipid metabolism, cell signaling, oncogenic proteins, anticancer therapy

## Abstract

Lipids, the primary constituents of the cell membrane, play essential roles in nearly all cellular functions, such as cell-cell recognition, signaling transduction, and energy provision. Lipid metabolism is necessary for the maintenance of life since it regulates the balance between the processes of synthesis and breakdown. Increasing evidence suggests that cancer cells exhibit abnormal lipid metabolism, significantly affecting their malignant characteristics, including self-renewal, differentiation, invasion, metastasis, and drug sensitivity and resistance. Prominent oncogenic signaling pathways that modulate metabolic gene expression and elevate metabolic enzyme activity include phosphoinositide 3-kinase (PI3K)/AKT, MAPK, NF-kB, Wnt, Notch, and Hippo pathway. Conversely, when metabolic processes are not regulated, they can lead to malfunctions in cellular signal transduction pathways. This, in turn, enables uncontrolled cancer cell growth by providing the necessary energy, building blocks, and redox potentials. Therefore, targeting lipid metabolism-associated oncogenic signaling pathways could be an effective therapeutic approach to decrease cancer incidence and promote survival. This review sheds light on the interactions between lipid reprogramming and signaling pathways in cancer. Exploring lipid metabolism as a target could provide a promising approach for creating anticancer treatments by identifying metabolic inhibitors. Additionally, we have also provided an overview of the drugs targeting lipid metabolism in cancer in this review.

## Introduction

Modifying cellular metabolism and energy production is a widely recognized characteristic of cancer cells (Hanahan and Weinberg, 2011). Various routes, processes, and proteins have a role in altering the cellular use of metabolites and chemicals to facilitate abnormal cell replication, spread from the original tumor, formation of new tumors, and evasion of the immune system. Comprehending the interaction between lipids, their metabolic processes, and associated signaling is paramount. Lipids consist of a wide variety of biomolecules with different compositions and functions, such as sterol and prenol lipids, fatty acyls, and glycerophospholipids, and they also play a significant role in tumors (Darwish et al., 2024). Lipids create the physical obstacles surrounding cellular organelles and defend the cell from the extracellular environment. Lipids can be used as substrates for biomass synthesis or stored for future oxidation to generate energy that supports cell movement and growth (Hopperton et al., 2014; Yao et al., 2016; Lin et al., 2017). Furthermore, lipids can directly bind to receptors, which allows them to trigger intricate signaling pathways that encourage cell growth and migration (Auciello et al., 2019; Wu et al., 2024). A high level of lipids or an imbalance between saturated and unsaturated fatty acids can disturb the body’s equilibrium and increase cellular strain. A characteristic of abnormal cell development and cancer progression involves alterations in lipid metabolism and signaling, which has only been recently recognized. The synthesis of phospholipids for cellular membranes is crucial in cancer cells and must be carefully regulated to meet other metabolic requirements. During stromal invasion, lipid compounds such as circulating free fatty acids (FFAs) might impact cancer cells. These molecules can supply extra substrates for cell development or significantly change cell signaling. When considering the changes that occur in the microenvironment as a consequence of obesity, these effects become much more significant (Boden and Shulman, 2002; Almoraie and Shatwan, 2024). Tumor cells can enhance the process of *de novo* lipogenesis, the absorption of fatty acids (FA), and the of fatty acid oxidation (FAO) to generate energy and accumulate lipids.

The crucial connection between the tumor microenvironment (TME) in facilitating and sustaining tumor growth is widely acknowledged. The complete population of immunological and stromal cells, in addition to cancer cells, must be considered when investigating the effects of lipids inside the TME (Wang JJ. et al., 2018; Corn et al., 2020). The cellular components and their interactions within the TME and the differences in cellular metabolism during different stages of cancer growth exhibit intricate and ever-changing characteristics (Yuan et al., 2022). In order to create more effective treatments, it is crucial to understand how different cell types affect cancer cell lipid metabolism or how lipids in the TME can affect them. Numerous research works have delineated the influence of lipid metabolism on the TME during the advancement of cancer (Yu X. et al., 2021). The interaction between a modified lipid metabolism and the TME can significantly influence other characteristics of cancer, such as through the involvement of lipid mediators such prostaglandin E2 (PGE2) and lysophosphatidic acid (LPA). As a result of their effects on tumor-promoting inflammation, angiogenesis, stromal cells, and immune cell compartment, these mechanisms can determine tumor progression and therapy resistance (Zelenay et al., 2015; Chiurchiù et al., 2018; Leuti et al., 2020). A better understanding of lipid metabolic reprogramming within the cancer milieu could discover new prognostic indicators and therapeutic targets for cancer treatment. Despite the importance of lipid metabolism in cancer, the link between lipid metabolism and carcinogenic signaling is not well understood. In a previous review, the authors presented studies on lipid metabolism in cancer progression and targeted only the main oncogenic pathways of deregulated lipid metabolism in cancer to a limited extent (Fu et al., 2020). A limited number of other publications highlight one or more cross-linked signaling pathways of dysregulated lipid metabolism in carcinogenesis processes in cancer (Hoxhaj and Manning, 2020; Tošić and Frank, 2021) and in a specific type of cancer (Hon et al., 2021). Moreover, the dysregulated mechanisms of cancer lipid metabolism have also been described, with no focus on the related cross-linked pathways or their therapeutic effects (Santos and Schulze, 2012; Sangineto et al., 2020). Thus, the objective of this review is focused on examining and combining several oncological pathways functionally associated with disrupted lipid metabolism. Ultimately, we proposed many potential therapeutic applications that specifically focus on addressing the modified lipid metabolism in cancer via targeting these signaling cascades and other potential targets.

## Altered lipid metabolism in carcinogenesis process

**FIGURE 1:**
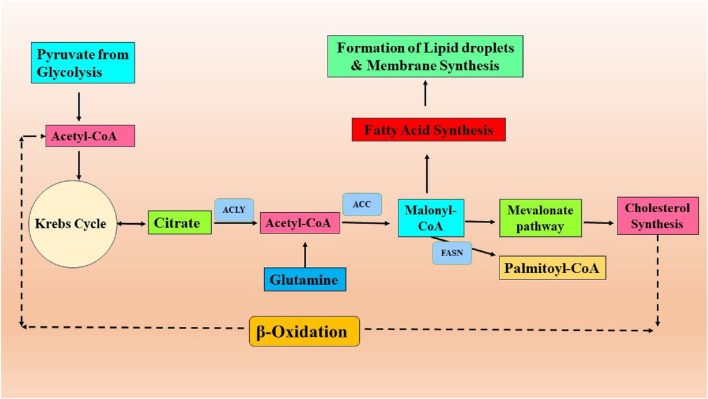
Lipid metabolic reprogramming in the process of cancer development.

Tumors possess remarkable adaptability, enabling them to flourish in unfavorable circumstances, ensuring their development and survival. The plasticity of cells is demonstrated through various metabolic phenotypes, which are influenced by genetic and epigenetic changes. These phenotypes are further impacted by the TME characteristics, including factors like nutrient and oxygen availability, pH levels, and cellular interactions (Strickaert et al., 2017). Lipid metabolism reprogramming is a critical factor in promoting the development and progression of tumors. Tumors can exhibit significant changes in their lipid profiles (Jin et al., 2023). A schematic illustration of abnormal lipid metabolism in cancer progression is presented in Figure 1.

Studies have long shown that certain cancers, including lung and breast cancer, exhibit an abnormal build-up of lipid droplets (LDs), which are associated with higher tumor grades (Medes et al., 1953). The aberration of lipid metabolism in cancer results from multiple intricate processes that contribute to the aggressiveness of tumors (Santos and Schulze, 2012). To begin with, cancer cells experience excessive stimulation of *de novo* lipid production or lipogenesis (Figure 1). More than 50 years ago, it was discovered that one of the critical characteristics of cancer cells is *de novo* lipid production (Medes et al., 1953). While non-transformed cells often obtain fatty acids from external food sources, cancer cells primarily rely on *de novo* fatty acid synthesis to produce lipids. This indicates that inhibiting this process could be an effective therapeutic strategy with few side effects. Lipogenesis necessitates the existence of acetyl-CoA, primarily obtained from the pyruvate derived from glucose, which enters the Krebs cycle (Figure 1). The Warburg effect in cancer can partially account for the excessive synthesis of fatty acids (Ocaña et al., 2020). Glucose provides carbon units for lipogenesis, although other carbon sources can be utilized if there is a shortfall in the glycolytic pathway. In low oxygen levels (hypoxia) conditions, glutamine can substitute glucose to generate α-ketoglutarate (αKG). This αKG molecule can then undergo reductive carboxylation through the action of isocitrate dehydrogenase 1 (IDH1) to make citrate. This process plays a crucial role in synthesizing lipids in cancer cells (Metallo et al., 2011). The somatic IDH1 R132H mutation, which generates the onco-metabolite 2-hydroxyglutarate, enhances *de novo* lipogenesis and FA oxidation in acute myeloid leukemia cells (Stuani et al., 2018). In contrast, acetate can also serve as a carbon source for lipid metabolism under low-nutrient circumstances. Acetate metabolism offers an alternative route for generating acetyl-coenzyme A (CoA) for lipogenesis, separate from converting citrate to acetyl-CoA. Cancer cells rely on certain members of the monocarboxylate transporter family to absorb external acetate. Once absorbed, acetate is transformed into acetyl-CoA by the action of acetyl-CoA synthetases (ACSS) (Walther et al., 2012; Schug et al., 2015). ACSS2 is overexpressed in human breast tumors, making them highly reliant on acetate for lipid production (Ligorio et al., 2021). These data emphasize the complex interconnection between glycolysis, glutaminolysis, acetate metabolism, and lipogenesis in cancer. An excessive amount of neutral lipids, such as triacylglycerols stored in LD, which build up in tumors to serve as a reserve of energy, are produced due to high levels of *de novo* FA synthesis. In addition, phospholipids are made to construct cancer cell membranes to meet the increased demand for cancer proliferation. Additionally, phospholipids are lipid messengers and intracellular signaling molecules in cancer, as reviewed by Cheng et al., 2016; Cheng et al., 2016). ATP citrate lyase (ACLY), acetyl-CoA carboxylase (ACC), and fatty acid synthase (FASN) are the most highly expressed lipogenic enzymes in several forms of cancer. Specifically, FASN has been identified as the crucial enzyme for lipogenesis, and its increased expression has been linked with an unfavorable prognosis in various cancer types (Kuhajda, 2006; Menendez and Lupu, 2007). Human malignancies, such as breast, colon, and prostate cancer, have elevated expression and activation of FASN, leading to an upsurge in the production of triacylglycerides (TG) deposited in lipid droplets (LDs). Therefore, targeting FASN in cancer is becoming increasingly intriguing (Mullen and Yet, 2015). Orlistat, a medication prescribed for treating obesity, has been found to specifically target FASN by blocking its thioesterase function, resulting in an anti-tumoral effect (Kridel et al., 2004). In addition, certain cancer types have been found to overexpress the pathway that produces mevalonate. This results in excessive cholesterol generation by converting acetyl-CoA by the enzyme 3-hydroxy-3-methylglutaryl-CoA (HMG-CoA) reductase. The presence of cholesterol in cancer cell membranes and abnormally elevated cholesterol levels in prostate cancer have been linked to the promotion of cancer growth (Xu et al., 2020). The sterol regulatory element-binding proteins (SREBP) transcription factors, which are triggered by the oncogenic PI3K/Akt/mTORC1 signaling cascade or cell cycle regulators, upregulate several enzymes involved in the fatty-acid and cholesterol-biosynthesis pathways in carcinoma (Ding et al., 2019). Monoacylglycerol lipase (MAGL) controls the amount of 2-arachidonoyl glycerol (2-AG) in the endocannabinoid system. Recent research has indicated that MAGL has a dual function, operating within the endocannabinoid system and exerting a significant influence on FFA levels to produce signaling lipids that promote tumor growth. MAGL, a serine hydrolase, is highly expressed in aggressive cancer cell lines, such as melanoma, ovarian, and breast cancer cell lines. It plays a role in the breakdown of monoacylglycerides into FFAs and glycerol (Nomura et al., 2010). Human prostate cancer cell lines that are not affected by androgens express higher levels of MAGL than androgen-dependent cell lines. Overexpression of MAGL in these cell lines has a dual effect on the endocannabinoid and fatty acid pathways, which promote the proliferation and aggressiveness of cancer cells (Nomura et al., 2011). MAGL is a constituent of a genetic signaling system associated with epithelial-mesenchymal transition (EMT) and the characteristics of stem cells in prostate cancer (Nomura et al., 2011).

Furthermore, in addition to the process of lipogenesis, it was noted that fatty acids (FAs), whether obtained from sources outside the cell or released from internal lipid reserves, can undergo oxidation within the mitochondria of cancer cells (Figure 1). In such circumstances, lipids serve as catalytic fuels, undergoing FAO or lipolysis to generate energy for cancer cells through ATP synthesis. In certain types of cancer, such as B cell lymphoma, the primary method of energy production is through mitochondrial FAO rather than relying on glycolysis (Caro et al., 2012). Three primary protein families participate in the cellular intake of fatty acids: the fatty acid translocase (FAT/CD 36/SR-B2) family, the membrane fatty acid binding protein (FABPm) family, and the fatty acid transport protein family (FATP) (Abumrad et al., 1999; Stremmel et al., 2001). Breast, ovarian, and colon malignancies have been shown to overexpress CD 36 (Lambrescu et al., 2024). There is strong evidence that CD 36 is involved in the aggressiveness of prostate cancer (Watt et al., 2019). Interestingly, studies have linked its overexpression to the epithelial-mesenchymal transition in hepatocellular carcinoma and, on the flip side, to leukemia survival (Farge et al., 2023; Deng et al., 2024). After entering the cytoplasm, FAs are attached to fatty acid-binding proteins (FABPs) and subsequently transformed into acyl-CoA. Acyl-CoA transportation into the mitochondria is facilitated by the protein carnitine palmitoyltransferase 1 (CPT1), which is frequently increased in cancer cells due to metabolic stress (Wang et al., 2018b). Carnitine palmitoyltransferase 1 (CPT1) is the primary enzyme that regulates the FAO. FAO occurs within the mitochondrial matrix and involves a cyclic catabolic process that produces nicotinamide adenine dinucleotide (NADH), flavin adenine dinucleotide (FADH2), NADPH, and ATP. In addition to mitochondria, FAO occurs in peroxisomes within cancer cells (Shen et al., 2020). Lipolysis and lipogenesis can co-occur in cancer cells (Carracedo et al., 2013). The cellular environment alters lipids’ metabolic process in cancer cells, namely, by the presence of adipocytes. Cancer-associated adipocytes serve as a significant supplier of external lipids for cancer cells. Research has shown a metabolic interaction between adipocytes and cancer cells, which causes the cancer cells to exhibit an aggressive phenotype (Mukherjee et al., 2022; Grunt et al., 2024). Cancer cells stimulate the release of FAs from nearby adipocytes through the secretion of soluble substances, including hormone-sensitive lipase and growth differentiation factor 15 (GDF15) (Bouche and Quail, 2023). The coexistence of cancer cells with adipocytes leads to the elevation of CD36 expression, resulting in an enhanced absorption of fatty acids by cancer cells. As a result, cancer cells absorb FAs generated by adipocytes. These fatty acids are then broken down in the mitochondria to provide the necessary energy for cancer cell growth, survival, spread, metastasis, and drug resistance (Kuo and Ann, 2018; Germain et al., 2020; Zakic et al., 2024). These findings emphasize the interaction between adipose tissue and cancer cells, which promotes cancer FAO and aggressiveness.

## Deregulated transcription factors of lipid metabolism involved in carcinogenesis

Lipid metabolic reprogramming is an important characteristic of tumors that contributes significantly to the development of cancer and resistance to therapy. Cancer cells rewire their metabolic pathways to produce a variety of essential signaling metabolites that influence signaling and epigenetic/transcriptional control, ultimately affecting cancer cell survival and proliferation in addition to energy and building blocks (Zhang et al., 2022). Gaining more insight into the mechanisms governing metabolic reprogramming in cancer could lead to the development of novel cancer targeting approaches. Recent research has indicated that dysregulated transcription factors have been detected in several types of human malignancies and have a substantial influence on lipid metabolism and signaling pathways in cancer. In this section, we examine the interplay between signaling and transcription factors in lipid metabolic reprogramming, and highlight the major transcription factors involved in lipid metabolic regulation.

## ChREBP

**FIGURE 2:**
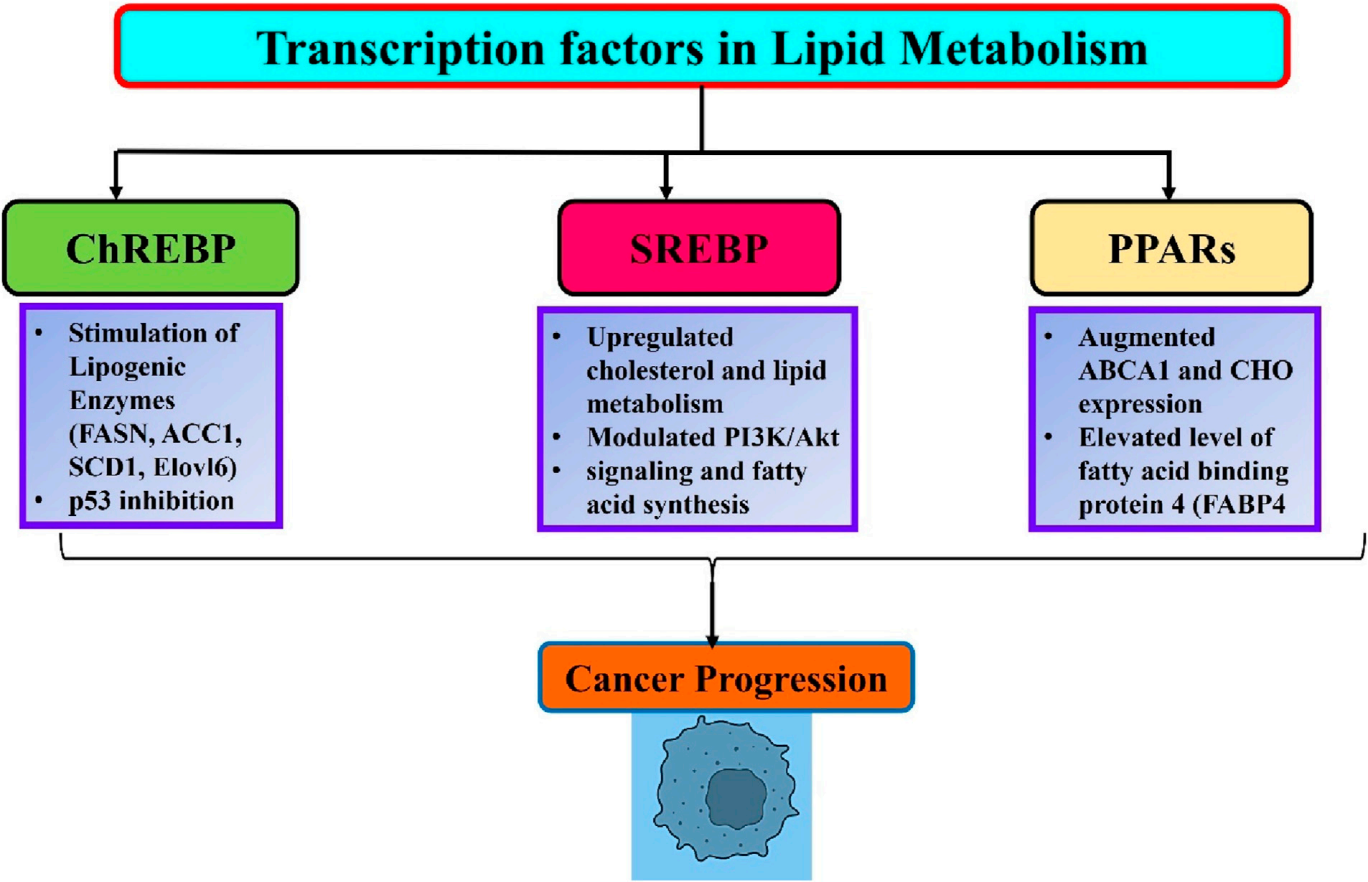
An overview of lipid metabolism related transcription factors and their role in cancer progression.

ChREBP is a glucose-regulated transcription factor that plays a crucial role in regulating enzymes involved in fatty acid synthesis (Uyeda et al., 2002). In response to glucose, ChREBP translocates from the cytoplasm to the nucleus and forms a complex with MLX1 to bind to the carbohydrate response elements of lipogenic genes. Nevertheless, the function of ChREBP is inhibited by adenosine monophosphate (AMP), ketone molecules, and cAMP (Ortega-Prieto and Postic, 2019). The ChREBP/MLX heterodimer controls glucose and lipid metabolism by regulating the expression of specific enzymes involved in glycolysis (PKLR, FK, GLUT2, GLUT4), gluconeogenesis (G6PC), and lipogenesis (FASN, ACC1, SCD1, Elovl6) (Ishii et al., 2004). (ChREBP) ChREBP mutant mice exhibit elevated hepatic glucose storage and reduced hepatic fatty acid levels compared with wild-type mice (Iizuka et al., 2004). ChREBP plays a significant role in the development of various cancers (Figure 2). ChREBP knockdown via siRNA effectively suppresses aerobic glycolysis and lipid production in colon cancer cells. Furthermore, inhibition of ChREBP triggers the activation of p53, leading to the interruption of the cell cycle and a decrease in the formation of colon cancer in living organisms. This suggests that ChREBP has an oncogenic role (Lei et al., 2020a). MLXIPL mRNA showed positive connections with glycolytic and lipogenic genes in chromatin immunoprecipitation analysis of human hepatocellular carcinoma (HCC) and breast cancer. The expression of glucose transporter 1 is dramatically elevated in individuals with hepatocellular carcinoma (HCC) and there is a positive correlation between its expression and ChREBP. Conversely, a contrasting relationship was observed between the ChREBP protein and pyruvate dehydrogenase kinase 2 genes. These genes are responsible for the deactivation of acetyl-CoA formation (Lei et al., 2020b). Cancer cells exhibit a preference for the transformation of pyruvate into acetyl-CoA as opposed to the buildup of pyruvate (Contractor and Harris, 2012). Given the positive correlation between ChREBP protein and tumor malignancy, our findings indicate that the carcinogenic function of ChREBP/MLXIPL arises from the conversion of glucose into fat (Airley et al., 2014).

## SREBPs

SREBP-1 functions as a transcription factor that controls lipid metabolism and reprograms tumor metabolism (Guo et al., 2014). SREBP controls the genes responsible for cholesterol production and fat breakdown (Horton et al., 2002). The levels and activity of SREBPs in normal tissues are closely regulated by endogenous sterol levels via a negative feedback mechanism. Nevertheless, cancer cells exploit the process of absorbing large amounts of glucose to produce fatty acids and cholesterol, which are essential for creating new cell membranes and lipid rafts (Vona et al., 2021). SREBP-1 has been recognized as a crucial factor in combining the flow of metabolism from glycolysis, which is influenced by PI3K/Akt signaling and fatty acid synthesis (Ricoult et al., 2016). SREBP-1 also controlled glutamine metabolism to promote lipid production (Figure 2) (Jhu et al., 2021). Moreover, research has demonstrated that SREBP-1 is upregulated in various types of malignancies and plays a role in promoting tumor growth (Cheng et al., 2018). Li CF. et al. (2017) found that by inhibiting the breakdown of SREBP, HCC progression can be reduced by regulating fatty acid (FA) and cholesterol metabolism. The anti-tumor capabilities of inhibiting the transfer of the SREBP-SCAP complex from the endoplasmic reticulum to the Golgi apparatus have been observed in many cancer cell lines, including HeLa, T98, and U2OS. Furthermore, the prevention of SREBP maturation not only hinders lipid metabolism but also disrupts the process of tubulin polymerization and construction of the mitotic spindle. This ultimately results in the reduced proliferation and migration of cancer cells (Gholkar et al., 2016). Overall, SREBP-1 is essential for the connection between oncogenic signaling and lipid metabolism (Figure 2).

## PPARs

PPARs, which are a subset of transcription factors, are part of the nuclear receptor family of proteins and have a significant impact on lipid metabolism. PPARs are composed of three primary isoforms, PPAR-α, PPAR-β, and PPAR-γ. These proteins exhibit varying levels of expression in various tissues, resulting in unique noncanonical activities in different cells. PPAR-α, which is present in adipose tissues, the liver, and the heart, controls fatty acid β-oxidation in fats. PPAR-β is widely expressed in several organs and plays a role in fat breakdown through β-oxidation (Brunetti et al., 2019). PPAR-γ expressed in adipose tissues promotes the development of adipose tissue and regulates fat production (Lodhi and Semenkovich, 2014; Ma et al., 2021). These proteins have been shown to control the function of other mitochondrial proteins, including carnitine palmitoyltransferase, citrate synthase, and cytochrome oxidases Hernandez-Quiles et al., 2021; Porcuna et al., 2021).

Moreover, significant connections between PPARs and tumors have been discovered in clinical and preclinical settings. The pan-cancer datasets of patients with 21 different types of cancer demonstrated that dysregulation of PPARs signaling disrupts several pathways related to lipid metabolism in tumor cells, which has a direct influence on the survival of patients (Chang and Lai, 2019. In addition, mice administered a PPARα agonist showed reduced body weight and a higher occurrence of liver cancer. Visible tumors and nodules with a diameter of approximately 11 mm were observed in the livers of mice treated with PPARα agonists (Peters et al., 1997). PPARδ promotes the formation of xenograft tumors derived from prostate cancer cells by modulating ATP-binding cassette transporter 1 (ABCA1) gene. ABCA1 is associated with efflux of cholesterol (CHO). PPARδ directly enhances the amount of ABCA1 mRNA and CHO expression in the cell membrane, leading to subsequent tumor growth (Her et al., 2013). PPARγ exhibits both tumor-suppressive and oncogenic effects in many types of cancers. PPARγ-mediated lipid production in lung cancer cells leads to a significant increase in mitochondrial reactive oxygen species stress, which plays a crucial role in suppressing tumor growth (Phan et al., 2017). Furthermore, pharmacological stimulation of PPARγ leads to an elevation in the production of fatty acid binding protein 4 (FABP4), which is followed by an increase in the amount of reactive oxygen species in lung cancer cells. The activation of PPARγ leads to the upregulation of FABP4 and lipoprotein lipase, which are associated with a more favorable prognosis in patients with lung and renal cancer (Hua et al., 2019). Conversely, the relationship between PPARγ and Nur77 has antagonistic effects on breast cancer. Nur77 engages PPARγ in the CD36 promoter and FABP4 to inhibit the transcription of these genes, thereby inhibiting the intake of fatty acids and the proliferation of cells. PPARγ directly interacts with Nur77 and enhances its ubiquitination of Nur77 through the action of the ubiquitin ligase Trim13, thereby exacerbating breast cancer (Yang et al., 2020). It is worth mentioning that dietary FAs have a direct binding effect on PPARs and imitate the actions of synthetic agonists that activate PPARs (Kliewer et al., 1997). PPARs detect fatty acid signals originating from dietary lipids, and act as lipid regulators to promote cancer progression (Figure 2). For example, a high-fat diet (HFD) stimulates the proliferation of tumor cells and facilitates the spread of colon cancer cells to the liver in mice. Nevertheless, deletion of PPARδ in mice effectively prevents the impact of a high-fat diet (HFD), as well as the expression of Nanog and CD44. This clearly indicates that PPARs play a crucial role in facilitating the tumor-promoting effect of HFD on cancer (Wang et al., 2019).

However, there are two more transcription factors of lipid metabolism FXR and LXR, their positive role in lipid metabolism reprogramming in cancer is still not validated yet. Therefore, we have discussed these two in detail, for more details please refer (Jeong et al., 2021).

## Lipids and oncogenic signaling

Interestingly, some data suggest that cancer signaling controls lipid production, and conversely, lipogenesis plays a role in regulating oncogenic signaling. In general, complex crosstalk synchronizes anabolic activities, DNA replication, and the cell cycle to supply the building blocks required for cell expansion and multiplication. The majority of fatty acids produced by FASN are incorporated into phospho- and glycolipids, which are important components of cell membranes (Zhou et al., 2003; Hilvo et al., 2011). Research has revealed that FASN contributes to the development of membrane lipid rafts, which in turn promote the clustering of signal protein complexes inside these crucial membrane microdomains. In addition, FASN enhances the production of membrane RTKs, including EGFR, ERBB2, and hepatocyte growth factor receptor/c-MET, in breast, ovarian, and prostate cancers, as well as non-Hodgkin lymphoma (Menendez et al., 2004a; Uddin et al., 2010). It also triggers the attraction of adaptor proteins such as GRB2 to EGF-activated EGFR (Wagner et al., 2017). As a result, when exposed to FASN inhibitory medications, the expression of EGFR and ERBB2 as well as the clustering of macromolecular signaling complexes at the membrane are compromised (Grunt et al., 2009). In conclusion, FASN is essential for the synthesis and development of membrane-signaling complexes (Swinnen et al., 2003). FASN can facilitate signal transduction by supplying signaling lipids including diacylglycerol, phosphatidylinositol, and phosphatidylinositol 3,4,5-trisphosphate (PIP3) (Benjamin et al., 2015). These secondary messengers play essential roles in mitogenic and pro-survival signaling. Furthermore, lipids produced by FASN can be utilized for the post-translational lipidation of proteins, which is necessary for proper intracellular localization and functioning of several signaling proteins. Additionally, lipidomic analyses have demonstrated that an overactive FASN enzyme increases the amount of saturated fatty acids compared to unsaturated fatty acids in the membranes of cancerous cells. This leads to a “saturated” lipid profile, which affects signaling processes and provides resistance against oxidative stress and cytotoxic drugs (Rysman et al., 2010). Since blocking or downregulating FASN stops the production of newly synthesized lipids, the processes of signal creation, propagation, and transmission at the cell membrane as well as downstream signaling cascades gradually deteriorate (Tomek et al., 2011).

Cancer is characterized by significant modification of phosphatidyl inositides (PIs) in cell membranes. PIs play a crucial role in defining the characteristics and functionality of membranes and also act as regulators of membrane trafficking (Vicinanza et al., 2008; Ooms et al., 2015). PI (3,4,5) P3 and PI (4,5) P2 play a significant role in tumor cell migration and metastasis, among other factors. They control cellular processes by enlisting, stimulating, or restraining proteins in the plasma membrane to influence actin dynamics, resulting in changes in cellular motility and metastatic ability. Phosphoinositide 3-kinase (PI3K) facilitates the formation of phosphatidylinositol-3,4,5-trisphosphate [PI (3,4,5) P3] from its precursor PI (4,5) P2. Increased levels of PI (3,4,5) P3 promote the localization of guanine nucleotide exchange factors (such as Vav and Tiam) of Rho GTPases to the cell membrane. This leads to the rearrangement of the cytoskeleton, which in turn improves cell migration and metastasis (Fritz and Kaina, 2006). Nevertheless, in breast cancer cells, a decrease in the amount of PI (4,5) P2 in the plasma membrane increases the ability of the cells to move and spread to other parts of the body (Sengelaub et al., 2016). The PI3K/AKT/mTORC axis affects different elements of metabolism and influences lipid anabolic metabolism signaling. PI3Ks are triggered by receptors on the cell surface and then convert the inputs into the buildup of the signaling lipid PI (3,4,5) P3. This lipid, in turn, enables the activation of many effectors such as the serine/threonine kinase AKT. AKT can add a phosphate group to ATP-citrate lyase, which in turn triggers the activation of several genes that play a role in the production of cholesterol and fatty acids (Manning and Cantley, 2007).

Sphingolipid metabolites, such as ceramide and sphingosine, are important regulators of angiogenesis, metastasis, and cell survival (Milstien and Spiegel, 2006). The biologically active lipid sphingosine-1-phosphate (S1P) is produced by the enzymes SPHK1 and SPHK2, and can be irreversibly destroyed by S1P lyase (SGPL1) or dephosphorylated by sphingosine phosphatase (Le Stunff et al., 2002). Five cell surface G protein-coupled receptors, collectively referred to as S1PR1-5 are the main mediators of autocrine or paracrine signaling by which S1P functions (Pyne and Pyne, 2010). SP1 can also bind to intracellular targets, including NF-κB and HDAC1/2 (histone deacetylases 1/2) (Maceyka et al., 2012). Increased S1P levels in oral squamous cell carcinoma (OSCC) were shown by Patmanathan et al. (2016) to be a result of decreased SGPL1 levels and increased SPHK1 transcription. S1P increased cell motility and invasion and shielded OSCC cells from cisplatin-induced cell death. Moreover, it has been shown that S1PR1 is intimately related to the ongoing activation of signal transducer and activator of transcription-3 (STAT3) and the expression of IL-6 in both tumor cells and the surrounding tissues. These elements are important in the development of tumor malignancies and their spread to distant locations (Lee et al., 2010).

When FAs are incorporated into cancer cell membranes, the membrane phases separate, cell contact decreases, surface adhesion increases, and tissues invade (Mitra et al., 2014). In an animal cancer model, Le et al. (2009) showed that mice fed a high-fat diet (HD) and an excess of plasma FFAs had an early initiation of a considerable number of CTCs and an elevated incidence of lung metastasis. The polarized distribution of cellular content results from the separation of the cancer cell membrane into many phases, caused by the high concentration of polyunsaturated FFAs in the blood plasma. The exposed cells had a strong resemblance to circulating tumour cells (CTCs) obtained from animals fed a high-fat diet. A class of tiny intracellular proteins known as FABPs binds lipids. FABP1–10 are the ten isoforms or variations of FABPs. Attaching and storing FAs and directing them to proper locations inside the cell-the plasma membrane, nucleus, endoplasmic reticulum, mitochondria, and peroxisomes-are functions of FABPs (Furuhashi and Hotamisligil, 2008). Inactive components of cell membranes and lipids form lipid rafts that facilitate the recruitment of signaling proteins and the interaction of proteins to initiate signal transduction pathways. As such, FABPs, which act as lipid chaperones, may improve signaling associated with metastasis by interacting with other proteins when they attach to membrane rafts. L-FABP, or liver fatty acid-binding protein, binds particularly to hydrophobic substances such as cholesterol and bile acids, as well as ligands such as long-chain fatty acids. Ku et al. (2016) found that the AKT/mTOR/P70S6K/4EBP1 and Src/FAK/cdc42 pathways are activated when L-FABP interacts with VEGFR2 in membrane rafts. The hepatocellular carcinoma cells to grow new blood vessels and migrate was improved by this activation, which also increased VEGF-A expression (Ku et al., 2016). Moreover, many FABP isoforms are strongly associated with the development of cancer metastasis. Increased expression of FABP3 or FABP4 in non-small cell lung cancer (NSCLC) negatively correlates with a more advanced tumor node metastatic stage and reduces the overall survival of patients with NSCLC (Tang et al., 2016). FABP5 inhibits EGF-induced signaling pathways linked to metastasis and partially inhibits the degradation of EGFR by proteasomes, thereby promoting the spread of triple-negative breast cancer. Moreover, FABP7 participates in fatty acid metabolism and may be a useful marker of metastatic melanoma (Rodic et al., 2014).

## Epigenetic modifications, lipid metabolism and oncogenic signaling

The interplay between cell metabolism and epigenetic gene regulation is now widely recognized, and cancer cells take advantage of this molecular connection (Carrer and Wellen, 2015). Recently, it was discovered that ACC can regulate global histone acetylation. This establishes a direct connection between anabolic metabolism and gene regulation through epigenetics (Galdieri and Vancura, 2012). Furthermore, free acetate, when converted to acetyl-CoA by acetyl-CoA synthetase (ACSS), functions as an epigenetic metabolite and enhances lipid synthesis under low oxygen conditions (Gao et al., 2016). As previously demonstrated, ACLY plays a crucial role in histone acetylation (Wellen et al., 2009). Lipids can serve as carbon sources and produce acetyl-CoA, which is utilized for histone acetylation. Furthermore, experimental data showed that ACLY preserves DNA methylation patterns in adipocytes and controls DNMT1 expression (McDonnell et al., 2016). DNA methylation relies on the presence of a methyl donor. Methionine adenosyltransferase metabolizes and converts the important amino acid methionine into S-adenosylmethionine (SAM). Every cell can produce S-adenosylmethionine (SAM). This is necessary as a preliminary step for the methylation of various substances such as DNA, RNA, proteins, lipids, and other small molecules (Varela-Rey et al., 2014). This process produces a demethylated compound called S-adenosylhomocysteine (SAH), which plays a role in cellular sulfur metabolism. S-adenosylmethionine (SAM) plays a crucial role as a co-substrate that facilitates the transfer of methyl groups by DNMTs. This process is essential for methylation of CpG islands located in the promoter regions of genes. In addition to this crucial epigenetic regulation process, researchers have also detected the methylation of certain lysines in the nucleosomal core histone proteins H3 and H4, with variations including mono-, di-, and tri-methylation. The presence of methylated lysine residues in histone proteins can either activate or inhibit gene transcription. This effect depends on the number of methyl groups added and the overall structural conditions around methylated lysine (Du et al., 2015). Interestingly, it has been found that there is an unanticipated correlation between the SAM:SAH ratio and methylation of either histone H3 and phosphatase PP2A or the membrane phospholipid phosphatidylethanolamine (PE). Existing evidence suggests that PE can function as a sink for cellular methyl groups, leading to the consumption of S-adenosylmethionine (SAM). Additionally, PE produces S-adenosylhomocysteine (SAH), which can be utilized in the transsulfuration pathway to convert methionine into cysteine. However, in cells that are not part of the liver, methyl is taken up by histone H3 and phosphatase PP2A, instead of PE (Ye C. et al., 2017). These observations indicate a reciprocal relationship between the methylation of membrane lipids, epigenetic control of histone proteins, and phosphorylation-dependent cellular signaling.

Dysregulation of the epigenome and growth signals is also considered as hallmark of cancer. A recent study has uncovered the pathways by which oncogenic signaling controls the regulation of epigenetic genes. Growth-promoting ligands interact with certain receptor tyrosine kinases (RTKs) and initiate RAS activation, which subsequently promotes downstream signaling pathways such as PI3K-AKT-mTORC1. PI3K activity stimulates cellular metabolism through multiple mechanisms. For example, AKT boosts glycolysis by upregulating the expression of glucose transporter 1 (GLUT1) on the cell membrane, activating phosphofructokinase, and promoting the interaction of hexokinases 1 and 2 with mitochondria. In addition, AKT stimulates the transcription of ACLY, which generates acetyl-CoA via the mTORC1-SREBP-1c pathway (Muñoz-Pinedo et al., 2012). In addition, AKT phosphorylates ACLY, resulting in stimulation of its catalytic activity (Potapova et al., 2000). In summary, AKT signaling promotes glucose absorption and metabolism through glycolysis, leading to the generation of significant amounts of citrate derived from the citric acid (Krebs) cycle. Furthermore, it enhanced the production of ACLY and initiated the enzymatic degradation of citrate into acetyl-CoA by ACLY. Therefore, AKT promotes the synthesis of acetyl-CoA in the cytosol. Acetyl-CoA is a crucial component necessary for histone lysine acetyl transferases to modify nucleosomal core histone proteins by acetylation and for FASN to produce lipids. It is generally accepted that a complex regulatory network that functions in numerous directions connects lipid metabolism, oncogenic signaling, and cancer epigenetics (Kinnaird et al., 2016).

## Key signaling pathways of altered lipid metabolism in cancer

While the significance of lipid metabolism concerning oncogenic pathways is acknowledged, the influence of lipid metabolism on the regulation of oncogenic signaling is gradually becoming apparent (Harayama and Riezman, 2018; Fu et al., 2020; Snaebjornsson et al., 2020). Cancer cells commonly exhibit excessive activation of oncogenic signaling pathways. Abnormal activation of critical pathways has been linked to changes in lipid metabolism in various types of cancer. In this subsequent part, we provide a concise overview of the latest significant breakthroughs in comprehending the role of lipid metabolism in promoting cancer growth and development through the modulation of diverse pathways.

## PI3K/Akt/mTOR signaling pathway

**FIGURE 3:**
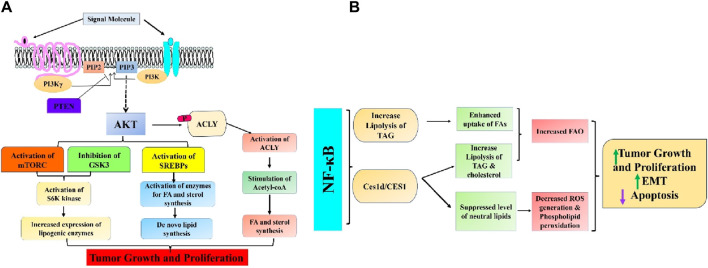
Intricate association between **(A)** PI3K/Akt/mTOR and **(B)** NF-κB signaling pathways and aberrant lipid metabolism in cancer.

Cancer cells frequently exhibit abnormal activation of lipid biosynthesis. Most cells in our body obtain the lipids they need by taking up fatty acids and lipoproteins from the bloodstream (Röhrig and Schulze, 2016). However, cancer cells activate *de novo* lipid biosynthesis to produce their lipids. This allows them to create cellular membranes and support rapid growth and multiplication (Menendez and Lupu, 2007). Sterols and fatty acids are both derived from cytosolic acetyl-CoA, which is synthesized either from citrate, an intermediate of the TCA cycle, through ATP citrate lyase (ACLY), or from acetate through acetyl-CoA synthetase. The PI3K–AKT pathway stimulates the production of new lipids through both post-translational and transcriptional mechanisms. AKT can facilitate the formation of new lipids by directly adding a phosphate group to ACLY, which enhances its activity and promotes the generation of cytosolic acetyl-CoA (Potapova et al., 2000; Berwick et al., 2002). This acetyl-CoA is then utilized to synthesize sterols, fatty acids, and protein acetylation reactions (Figure 3A). Previous studies have indicated that the AKT-ACLY pathway enhances tumor development and has a widespread impact on histone acetylation (Lee et al., 2014; Carrer et al., 2019). Besides its role in regulating cancer development through the PI3K-AKT pathway, ACLY is often excessively expressed in different human cancers. Blocking ACLY activity reduces the growth of cancer cells both in laboratory settings and living organisms, suggesting that this enzyme could be a promising target for cancer treatment (Hatzivassiliou et al., 2005; Zu et al., 2012). Due to its crucial involvement in lipid production, ACLY inhibitors, initially designed for metabolic conditions like hypercholesterolemia and type 2 diabetes, are now being explored as potential treatments for cancer (Granchi, 2018; Ray et al., 2019). The AKT signaling pathway also stimulates *de novo* lipid synthesis by activating the sterol regulatory element-binding protein (SREBP) family of transcription factors (SREBP1a, SREBP1c, and SREBP2). These transcription elements activate the expression of almost all enzymes involved in fatty acid and sterol synthesis, particularly ACLY (Porstmann et al., 2005; Li N. et al., 2023). The SREBPs are present as inactive transmembrane proteins in the endoplasmic reticulum (ER) and need to be transported to the Golgi apparatus for proteolytic processing to become active. The processing of SREBP results in the release of the N-terminal segment of the protein, which acts as the mature and active form. This segment then moves to the nucleus and starts the transcription process by binding to sterol response elements present in the promoter region related to lipid production and the production of NADPH, which is necessary for lipid synthesis. AKT triggers the activation of SREBP through a minimum of two subsequent pathways. Studies have demonstrated that mTORC1 promotes the processing and movement of SREBPs into the nucleus through various suggested mechanisms. This results in the stimulation of genes involved in lipid production and an elevation in the synthesis of new lipids in the liver in response to insulin or in cancer cells following the activation of oncogenic PI3K or Ras (Yecies et al., 2011; Owen et al., 2012; Ricoult et al., 2016). After processing, the fully developed active form of SREBP is directed towards ubiquitin-dependent degradation through GSK3-mediated phosphorylation (Kim et al., 2004; Sundqvist et al., 2005). Therefore, the activation of AKT signaling can enhance the processing of SREBP by activating mTORC1, and it can also increase the stability of the processed active SREBP by inhibiting GSK3. Recently, it has been reported that MYC collaborates with SREBP to stimulate lipogenesis and facilitate cancer proliferation (Gouw et al., 2019). Notably, many messenger RNAs stimulated by SREBP are controlled by the serine/arginine-rich (SR) protein family of splicing factors. Stimulation of mTORC1 signaling has been observed to promote the splicing of these mRNAs that encode lipogenic enzymes. This stimulation occurs through activating S6 kinase 1 (S6K), a downstream target of mTORC1, which phosphorylates and triggers SR protein kinase 2 (SRPK2). As a result, the SR proteins are phosphorylated and activated, leading to enhanced splicing of the mRNAs (Lee et al., 2017). Several factors and enzymes that play a role in the production of new lipids, such as SREBPs, SRPK2, and the lipogenic enzymes activated by SREBPs, have been observed to be increased in various types of cancer. These factors and enzymes are considered potential targets for cancer treatment. Collectively, these transcriptional programs, in conjunction with the aforementioned post-translational regulatory mechanisms, effectively amplify lipogenesis in cancer cells stimulated by oncogenic PI3K-Akt-mTOR signaling (Urbanski et al., 2018).

## NF-kB signaling

NF-κB, a crucial transcription factor implicated in controlling the inflammatory response, is also a significant signaling pathway for cell survival that is frequently hyperactivated in numerous cancer types (Zinatizadeh et al., 2020). New research has demonstrated that NF-κB, through controlling metabolic programs affecting glycolysis, glutaminolysis, OXPHOS, and other metabolic pathways, is essential for tumor cells’ response to microenvironments deficient in nutrients. Preliminary findings in tumor cell lines indicated that NF-κB has a role in the development of colorectal cancer (CRC) by facilitating metabolic adaption to energy stress (Mauro et al., 2011). A recent study showed that the NF-κB-dependent adaptative mechanism is intrinsically connected to the regulation of lipid metabolism. This was supported by the observed changes in lipidomic profiles of RelA/RELA-deficient mice embryonic fibroblasts and human CRC cell lines (Capece et al., 2021). Recently, a study in *Drosophila melanogaster* revealed evidence of a historical link between nutrient imbalance and lipid metabolism. The link between these two factors is facilitated by NF-κB, a protein that has a vital function in the body’s reaction to fasting. NF-κB restrains the breakdown of triacylglycerol lipids by repressing the gene that encodes adipose triglyceride lipase (ATGL)/Brummer in the fat tissue. This metabolic process is recognized for maintaining cellular triacylglycerol reserves, the primary energy source in multicellular organisms (Molaei et al., 2019). The stimulation of NF-κB in human colorectal cancer cell lines during nutrient deprivation can enhance triacylglycerol lipolysis, which in turn mobilizes endogenous FFAs from lipid droplets and fuels FAO and OXPHOS during energy supply, in contrast to the result of this ancient adaptive mechanism in insects. Hence, the suppression of NF-κB hindered the breakdown of lipids during periods of low energy, reducing the flow of oxidative phosphorylation and the synthesis of ATP in the mitochondria, finally leading to metabolic crisis and cell death (Capece et al., 2021). Through integrating a metabolomic and transcriptome analysis, the researchers discovered carboxylesterase 1d (Ces1d), the mouse equivalent of human CES1. They found that Ces1d is a lipase regulated by NF-κB and has a crucial role in increasing the survival and adaptation of cancer cells to limited nutrition in aggressive human colorectal cancer (CRC). Furthermore, the metastasis-prone CRC consensus molecular subtype (CMS)4 exhibited higher CES1 expression and NF-κB activity, which were linked to inflammation, obesity, the epithelial-to-mesenchymal transition (EMT), and poorer clinical outcomes for CRC patients (Capece et al., 2021). Increased CES1 expression was also shown to be associated with reduced survival rate in overweight individuals with colorectal cancer (CRC) but not in non-overweight patients. This suggests that the NF-κB/CES1-dependent breakdown of fat may play a role in the clinical progression of CRC, specifically in obese patients. Ces1d/CES1 increased triacylglycerol and cholesterol ester lipolysis to transport endogenous FFAs and fuel FAO to fulfill the energy needs during nutrient deprivation. It prevented harmful neutral lipid accumulation that causes ROS generation and phospholipid peroxidation, which causes apoptosis and ferroptosis. Hence, the inhibition of Ces1d/CES1 through genetic or pharmacologic means resulted in the death of colorectal cancer (CRC) cells under conditions of nutrient deprivation in laboratory settings and reduced CRC growth in animal models. These findings indicate that CES1 could be a potential target for treating CRC. Ultimately, through modulating many oncogenic signaling pathways, NF-κB activation stimulates lipid metabolism and plays a vital role in cancer cell survival under metabolic stress (Figure 3B).

## STAT3 signaling

**FIGURE 4:**
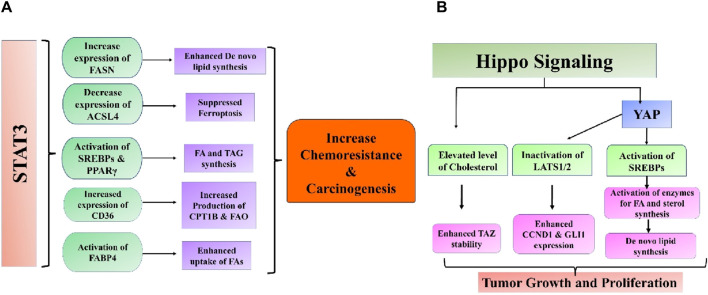
Intricate association between **(A)** STAT3 and **(B)** Hippo signaling pathways and aberrant lipid metabolism in cancer.

Cancer cells alter their intracellular lipid metabolism to optimize the use of available resources, in addition to being impacted by adipose tissue through paracrine and endocrine processes. Remarkably, STAT3 may fulfill various functions in this particular process (Tošić and Frank, 2021). Lipids serve multiple purposes for cancer cells in environments with limited nutrition: they can be used as an alternative energy source, a component of cell membranes to facilitate fast division and proliferation, a defense mechanism against excessive oxidative stress, and a building block for lipid-based hormones that promote cancer cells’ pathological growth (Vidavsky et al., 2019). Endogenous lipids have been linked to various cellular processes, including growth, differentiation, inflammation, autophagy, cell death, and membrane formation (Alizadeh et al., 2023). Therefore, changes in the levels of proteins that control the production, breakdown, and storage of lipids have been extensively documented in cancerous cells (Lodhi and Semenkovich, 2014). An example is when breast malignancies have increased HER2/neu, often followed by an amplification of the peroxisome proliferator-activated receptor gamma (PPARγ) binding protein (PBP) gene. This may be because these genes are located close to each other on chromosome 17q12–21 (Kauraniemi et al., 2001). Curiously, the STAT3 gene is also situated near these genetic locations, specifically at chromosome 17q 21.2. Both the overexpression of PBP and the activity of STAT3 boost the transcriptional activity of PPARγ, a significant lipid metabolism regulator and an essential mediator of adipogenesis and adipocyte differentiation (Wang C. et al., 2009; Kourtidis et al., 2009). Moreover, both the process of synthesizing fatty acids (controlled by the enzyme FASN) and the process of FAO are frequently increased in cancerous cells (Figure 4A) (Yoshii et al., 2015; Wang et al., 2018c). FASN-mediated *de novo* fatty acid synthesis and anabolic-driven growth, survival, and migration can be initiated by activating PI3K/AKT/mTOR and MAPK and by overexpressing HER/neu (Fhu and Ali, 2020). Additionally, it has been demonstrated that STAT3 activation increases the expression of FASN (Gao et al., 2020). The connection between FASN inhibition and trastuzumab resistance reversal in HER2-positive breast tumors has therapeutic potential (Vazquez-Martin et al., 2007). Furthermore, the breakdown of fatty acids (FA) through FAO promotes the growth of tumors by supplying ATP to meet the energy requirements of cells during metabolic stress caused by a lack of glucose (Qu et al., 2016). Activation of leptin, IL-6, or STAT3 through other mechanisms stimulates the production of carnitine palmitoyltransferase 1B (CPT1B), an enzyme that controls the rate of fatty acid oxidation (Koo et al., 2019). This pathway plays a key function in the self-renewal of breast cancer stem cells (BCSCs) and enhances their ability to survive chemotherapy. The process of FAO generates NADH and FADH2, which can decrease oxidative stress and increase ATP synthesis through the electron transport system (Koundouros and Poulogiannis, 2020). FAO also produces acetyl-CoA, which can be used for energy production via the Krebs cycle or for the generation of fatty acids and protein acetylation. These processes have the potential to stimulate tumor growth. Conforming to this correlation, the addition of acetyl-CoA partially counteracted the impacts of suppressing STAT3 on the growth and viability of breast cancer stem cells (BCSCs). Furthermore, the augmentation of FAO plays a crucial role in the emergence of chemoresistance in several malignant systems, such as AML and breast, lung, and stomach malignancies. Inhibiting this route hinders the abnormal proliferation of cancer cells and restores their sensitivity to chemotherapy (Li J. et al., 2013; He et al., 2019). Another possible strategy for utilizing the lipid properties mediated by STAT3 for therapeutic purposes is to differentiate between cancer cells and normal cells, which often have minimal STAT3 activity. The activation of STAT3 and subsequent malignant transformation of mammary epithelial cells leads to a substantial decrease in the cellular levels of N-acyl taurine and arachidonic acid (Tošić et al., 2021). Given the significance of both these compounds in plasma membrane modeling, an investigation was conducted to determine if this characteristic might be effectively utilized in innovative nanoparticle drug delivery systems for therapeutic purposes. Nanoparticles coated with poly-L-glutamic acid had a strong affinity for cells with aberrant STAT3 activity. When these particles were used to carry cytotoxic drugs, they specifically generated higher levels of apoptosis in these cells. A recent paper highlighted the significance of lipid architecture in determining the metastatic potential of cancer cells and its impact on cell survival and proliferation (Jin et al., 2020). This study discovered that cancer cells with a high ability to spread to other parts of the body had a larger amount of cholesterol, phosphatidylcholine (PC), and sphingomyelin (SM) in their cells. On the other hand, these cells have lower amounts of triacylglycerol. This finding aligns with the patterns observed in cellular TAG and SM levels after STAT3 activation in breast cancer cells, as reported by previous studies (Tošić et al., 2021). Jin et al. (2020) also demonstrated that various metabolic regulators significantly impacted the ability of breast cancer cells to develop brain metastases. These regulators include sterol regulatory element-binding protein 1 (SREBP1), which plays a crucial role in the synthesis of fatty acids (FA) and triglycerides (TAG), as well as the FA transporter CD36 and FA binding protein FABP6 (Jin et al., 2020). Interestingly, a correlation was observed between the activity of STAT3 and the mRNA expressions of SREBP1 and CD36 in several cellular and animal models. The expression of SREBP1, driven by high glucose, is dependent on the tyrosine phosphorylation of STAT3 (Guillet-Deniau et al., 2004), and this requirement is significantly reduced when STAT3 is silenced (Chen et al., 2018). Likewise, the excessive production of an always active version of STAT3 substantially increased the expression of CD36, and the signaling pathway, including JAK2/STAT3, was necessary for the induction of CD36 expression by IL13 (Yakubenko et al., 2013; Su et al., 2020). The data indicate that the ability of STAT3-driven malignancies to spread to other parts of the body may be partly due to its diverse impacts on lipid metabolism. However, additional research is required to understand these interesting connections’ underlying mechanisms fully. STAT3 was discovered to hinder cell ferroptosis by repressing the production of acyl-CoA synthetase long-chain family member 4 (ACSL4), an enzyme that promotes ferroptosis by increasing the presence of long polyunsaturated fatty acids in cell membranes (Ding et al., 2023). It was also demonstrated that STAT3 can collaborate with androgen receptor (AR) to stimulate the expression of cell cycle-related kinase (CCRK), thereby augmenting the tumorigenicity of liver cancer. Functionally, it has been demonstrated that CCRK enhances the presence of the fully developed version of SREBP1 in the nucleus through GSK3β/mTORC1 signaling, leading to an augmentation in the production and absorption of new lipids (Sun et al., 2018). STAT3 was discovered to enhance the intake of fatty acids (FA) in ovarian cancer, leading to the activation of FABP4, which in turn supported the proliferation of cancer cells (Yu et al., 2020).

## Hippo signaling pathway

It has been observed that *de novo* lipogenesis is regulated by crucial elements of the hippo cell signaling system. Within the endoplasmic reticulum (ER), LATS2 interacts with SREBP-2, causing it to be sequestered and preventing its transport from the ER to Golgi (Lee et al., 2022). Furthermore, YAP can serve as a coactivator with SREBP-1c and SREBP-2 in the nucleus to enhance the expression of genes linked with lipid synthesis (Shimano and Sato, 2017; Shi et al., 2019). The Hippo signaling pathway serves as a key upstream regulator of lipid production and a mediator for liver maintenance, crucial for the liver’s recovery from metabolic stress damage. Lipids can regulate the Hippo signaling pathway, potentially leading to the development of liver diseases such as nonalcoholic steatohepatitis (NASH) progressing to HCC or fibrosis (Wang et al., 2020). Non-alcoholic fatty liver disease (NAFLD) is marked by excessive fat buildup leading to elevated cholesterol levels and is recognized as a significant contributor to the sustenance and survival of cancer cells (Novak et al., 2013). High cholesterol levels have been identified to reduce TAZ phosphorylation through LATS1/2 by activating RhoA, which inhibits LATS1/2. TAZ expression increased in fibrotic NASH livers compared to normal or non-fibrotic ones. The study also indicated that high cholesterol levels could enhance TAZ stability, leading to the promotion of NASH fibrosis (Wang et al., 2016). Inactivating LATS1/2 may promote the progression from NASH to HCC, as indicated by another study. Within NASH-HCC, the junction protein JCAD linked to obesity interacts with LATS2, hindering its kinase activity by binding to the kinase domain but not affecting the connection between LATS2 and MOB1. By inhibiting kinase activity, YAP phosphorylation level, cellular location, and activity are directly affected, leading to the expression of target genes, including CCND1 and GLI2, which stimulate cell proliferation (Rinella, 2015; Ye J. et al., 2017). Lipid uptake is a crucial activity that influences lipid availability to cells and is increased in various malignancies, especially in metastasis-initiating cells and NAFLD. Fatty acid uptake occurs through fatty acid translocase CD36 and members of the FABP family (Pascual et al., 2017; Feng et al., 2022). Consistently, increased CD36 expression has been seen in NAFLD patients and cells that initiate metastasis. CD36 facilitates the intake of oxidized LDL, which can provide FFAs by enhancing lipophagy, a specialized type of autophagy that degrades lipid droplets. Lysophosphatidic acid (LPA) levels increase due to lipophagy, activating YAP (Tian et al., 2019). SAV1 deletion upregulates CD36 mRNA expression in PTEN-deficient livers by inhibiting YAP, an upstream negative regulator. CRABP2, a lipid transporter belonging to the FABP family, is suggested to influence the Hippo signaling pathway in triple-negative breast cancer through its interaction with estrogen receptor alpha (ER). The association between CRABP2 and LATS1 inhibits the ubiquitination of LATS1, leading to the activation of Hippo signaling in ER-positive tumors. This process helps to prevent the invasion and spread of cancer cells. Ubiquitination of LATS1 enhances the invasion and metastasis of ER-negative malignancy. It is not apparent how ER expression influences the function of CRABP2. The correlation between lipid absorption and the Hippo signaling pathway is intricate yet crucial in advancing aggressive cancer (Feng et al., 2019) (Figure 4B).

## Wnt/β-catenin signaling

**FIGURE 5:**
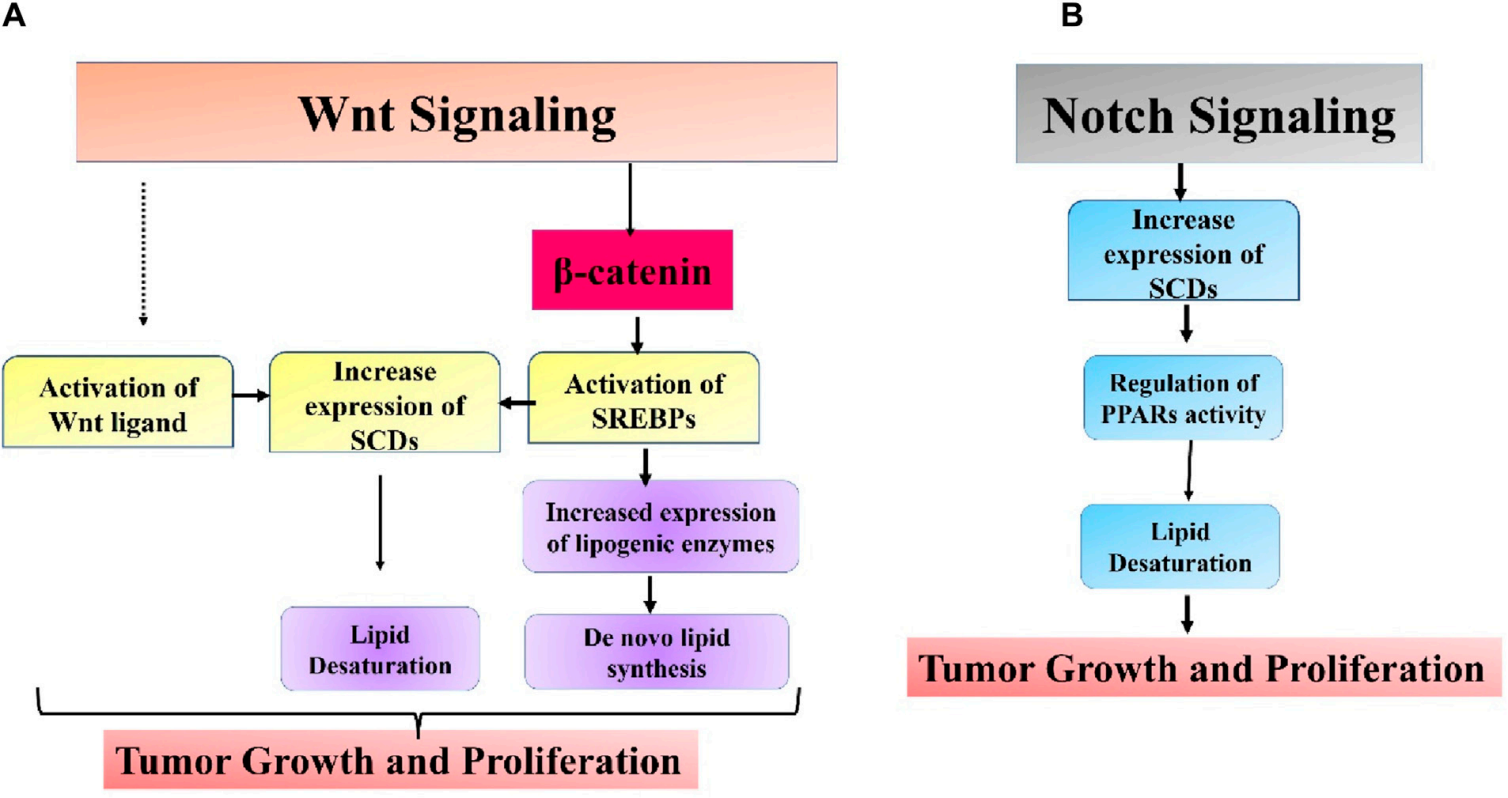
Intricate association between **(A)** Wnt and **(B)** Notch signaling pathways and aberrant lipid metabolism in cancer.

It has also been found that Wnt signaling is involved with the process of cancer lipogenesis (El-Sahli et al., 2019). Glutamine can be redirected to reductive carboxylation to produce α-ketoglutarate, which generates acetyl-CoA for *de novo* lipogenesis. *De novo* lipogenesis provides high energy levels and building blocks for critical cellular components to expand cells. Deviant lipid levels in cells are associated with the advancement of cancer in several types of malignancies. In breast cancer, there is an increased buildup of FFAs, cholesterol esters, and phospholipids, which have been proven to enhance tumor growth and invasion by cancer cells. Tumors rich in cholesterol ester were linked to higher rates of breast tumor growth and tissue death. Linoleic acid was demonstrated to stimulate breast cancer cell movement through plasminogen activator inhibitor-1 (PAI-1) and SMAD4 (Byon et al., 2009). Yao et al. (2018) discovered in a recent study that canonical Wnt signaling via MYC facilitates the transformation of triacylglycerol into phospholipids and enhances the presence of unsaturated fatty acyl groups in phospholipids. Cancer cells heavily rely on lipid metabolism remodeling to incorporate unsaturated fatty acids into their cell membranes for maintenance, energy storage, and signaling purposes. Unsaturated fatty acids are associated with the stem-like traits in ovarian cancer (Mukherjee et al., 2017). Reducing β-catenin in breast cancer cells led to a decrease in critical lipogenic enzymes such as citrate carriers, ACC, and FASN. This highlights the involvement of the Wnt/β-catenin pathway in *de novo* lipid synthesis (Vergara et al., 2017). Mounting data has emphasized the importance of lipid metabolism in cancer stem cell survival and upkeep. Subtle discrepancies exist in the lipid metabolism changes between CSC and non-CSC groups. In breast cancer, the enzyme carnitine palmitoyltransferase I (CPT1), responsible for fatty acid β-oxidation, is found to be higher in the CSC (cancer stem cell) population compared to the non-CSC population (Wang Y. et al., 2018). Stearoyl-CoA desaturases (SCDs) could be the crucial link between Wnt signaling and lipid metabolism in these CSCs. SCDs speed up the process of lipid desaturation, and there is increasing evidence pointing to SCDs as a characteristic feature of CSCs. The relationship between the Wnt pathway and SCDs is firmly established. β-catenin has been shown to enhance the sterol regulatory element binding protein 1 (SREBP-1)-associated expression of the primary types of SCD (SCD1 and SCD2) in CSCs. Suppression of SCD increased the levels of β-catenin expression, which was reversed by adding monounsaturated fatty acids (MUFAs) produced by SCDs (Lai et al., 2017). In another investigation, MUFAs were essential for Wnt ligand synthesis and secretion. SCD1 was demonstrated to control the Hippo/YAP pathway, a crucial cancer stem cell-related oncogenic pathway, partly through Wnt signaling. Simultaneous blocking of Wnt and YAP has been demonstrated to slow down the progression of triple-negative breast cancer in both mesenchymal and epithelial forms (Rios-Esteves and Resh, 2013; Sulaiman et al., 2018). Collective research indicates that Wnt pathway may play a role in lipid metabolic changes in cancer cells, and targeting both pathways could be more efficient in addressing CSCs (Figure 5A).

## Notch signaling

The Notch pathway is a highly conserved mechanism for transmitting signals strongly associated with biological processes like tumor spread and immune evasion (Pandey et al., 2023a; Pandey et al., 2023b). The Notch signaling system can control the expression of peroxisome proliferator-activated receptor α and lipid oxidation genes to maintain lipid and redox equilibrium in lipid metabolism. Targeting SCD1-dependent lipid desaturation in colon cancer preferentially kills colon CSCs by blocking Notch signaling (Yu Y. et al., 2021). A recent study found that Notch controls lipid metabolism, which is associated with the beginning of tumor formation. Notch signaling-induced changes in lipid metabolism are crucial for the development of liposarcomas. Adipocytes that activate the Notch pathway experience dedifferentiation, leading to lipid metabolic failure and the eventual development of liposarcoma. Blocking Notch signaling can decrease lipids’ absorption and fatty acid oxidation. A possible mechanism was proposed involving Notch-induced suppression of PPARγ ligands synthesis, which are crucial regulators of lipid balance in adipocytes. Administering a synthetic PPARγ supplement partially reversed the transformation of adipocytes produced by Notch activation (Bi et al., 2016). Endothelial cells have been found to control the production of endothelial lipase, an enzyme associated with fatty acid transport through the vessel wall through the Notch signaling pathway (Jabs et al., 2018). The overall impact of Notch signaling in cancer remains mainly uninvestigated, and additional research is needed to ascertain if other molecular pathways could play a role in Notch-induced changes in lipid metabolism in cancer (Figure 5B).

## Therapeutic intervention targeting lipid metabolism in cancer

FASN inhibitors have been extensively studied, and one of the compounds that has progressed to clinical testing is TVB-2640. TVB-2640 has notable anticancer effects in several types of carcinoma, such as lung, ovarian, and breast cancers (Falchook et al., 2021). C75, a chemical that targets FASN, enhances fatty acid oxidation and induces cell death during S phase in breast cancer cells (Menendez et al., 2004b). The FAS inhibitor C75 reduced lipid droplet formation, PGE2 synthesis, and cell proliferation in CACO-2 cells, indicating that lipid droplets serve as regulatory reservoirs for prostaglandin formation and play a role in mediating inflammatory pathways crucial for cancer cell metabolism (Accioly et al., 2008). Lipid metabolism was investigated in a transgenic mouse model of medulloblastoma, including a mutated smoothened allele that disrupts the sonic hedgehog pathway (Bhatia et al., 2011). Approximately two-thirds of the mice acquired MB. The tumor area showed higher levels of FASN expression, increased production of new fatty acids, and reduced fatty acid breakdown. The increased levels of FASN were likewise associated with the levels of the transcription factors E2F1 and MYCN (Bhatia et al., 2011). Animals with medulloblastoma, when treated with the fatty acid synthesis inhibitor C75, exhibited improved survival, reduced lipid synthesis, and decreased expression of proliferation markers MYCN and cyclin D2. Acetyl CoA carboxylase was also identified as being improperly controlled in this mouse model (Bhatia et al., 2011).

Cerulenin was the first naturally occurring, non-competitive inhibitor of FAS that has been found. Cerulenin undergoes covalent bonding with a cysteine residue located in the active site of the ketoacyl synthase (KS) domain of the fungal FAS enzyme. This interaction leads to substantial alterations in the conformation of the active site (Mallick et al., 2023). Cerulenin inhibits the activation of estrogen receptors in ovarian cancer, hinders the spread of colon cancer to the liver, and triggers cancer cell death in both laboratory and living organisms (Menendez et al., 2004a; Shiragami et al., 2013). Orlistat is an FDA-approved medication for managing obesity authorized in 1999. Orlistat triggers endoplasmic reticulum stress, leading to increased apoptotic cell death in prostate, breast, and colon cancer cells (Menendez et al., 2005; Little et al., 2007). Preclinical studies are currently being conducted on the utilization of ACC inhibitors. Soraphen A has demonstrated anti-cancer effects by blocking the ACC enzyme in cancer cells. Beckers et al. (2007) discovered that Soraphen A effectively suppressed fatty acid production in LNCaP and PC-3M prostate cancer cells at extremely low concentrations, leading to an enhanced process of β-oxidation of fatty acids. This resulted in a reduction in phospholipid levels within cancer cells, ultimately hindering their growth (Beckers et al., 2007). ND-646 is an allosteric inhibitor of ACC that hinders the formation of non-small cell lung carcinoma in both laboratory settings and living organisms (Svensson et al., 2016). TOFA, an allosteric inhibitor of ACC, triggers cell death by releasing pro-apoptotic proteins from the mitochondria in breast, colon, lung, and ovarian cancers (Wang D. et al., 2009; Guseva et al., 2011; Li S. et al., 2013). A939572, an SCD1 inhibitor, triggers endoplasmic reticulum stress and induces apoptotic death in renal cell carcinoma (RCC) (von Roemeling et al., 2013). A939572 inhibits phosphorylation of the PI3K/Akt pathway and markedly reduces the viability of lung cancer cells in living organisms (She et al., 2019). Another potential strategy involves targeting the cholesterol synthesis. Statins are inhibitors of β-hydroxy β-methylglutaryl-CoA reductase (HMGCR), and have demonstrated promising results in laboratory and living organism studies. Statins hinder lipid metabolism and promote cell survival in different types of cancers, such as colon, pancreas, liver, breast, prostate, bladder, lung, and skin cancers (Apostolova et al., 2016; Ahmadi et al., 2020). Statin use has been shown to lower the occurrence and reappearance of multiple types of cancer in 3129 human epidemiologic studies, including bladder, breast, colon, kidney, lung, skin, pancreatic, and prostate cancers (Cauley et al., 2006; Cao et al., 2023; Takada et al., 2023; Wang et al., 2023; Zhang et al., 2024). Simvastatin, a cholesterol synthesis inhibitor, blocks the Akt pathway and induces apoptosis in prostate cancer cells (Zhuang et al., 2005). A preliminary phase II trial in NSCLC investigated the possibility of simvastatin enhancing the effectiveness of gefitinib in a specific subgroup of gefitinib-resistant patients (Han et al., 2011). Furthermore, a recent study conducted in patients with locally advanced breast cancer indicated that the combination of simvastatin and neoadjuvant chemotherapy resulted in enhanced therapeutic responses (Yulian and Siregar, 2021). Nevertheless, a phase III clinical trial conducted on patients with advanced gastric cancer found no significant benefit of combining simvastatin with capecitabine-cisplatin compared to a placebo in combination with capecitabine-cisplatin (Kim et al., 2019).

Another strategy for targeting lipid uptake focused on CD36 and FABPs. ABT-510, a synthetic analog of thrombospondin-1, is presently undergoing phase I clinical trials for the management of glioblastomas, melanomas, and renal cell carcinomas (Ebbinghaus et al., 2007; Markovic et al., 2007; Nabors et al., 2010). ABT-510 binds to CD36 and triggers the expression of the death receptor FAS (Isenberg et al., 2008). Al-Jameel et al. (2019) developed a potent recombinant FABP5 inhibitor, dmrFABP5. FABP5 interacts with and carries medium- and long-chain fatty acids in the nuclei of cancer cells. dmrFABP5 is produced by substituting three amino acids in FABP5, resulting in loss of its capacity to bind fatty acids. dmrFABP5 significantly inhibited the proliferation, migration, and invasion of prostate cancer cells (Al-Jameel et al., 2019). When exposed to chemotherapeutic drugs such as Docetaxel or Cabazitaxel, two advanced synthetic inhibitors, Stony Brook fatty acid-binding protein inhibitor 102 (SBFI-102) and SBFI-103, were found to effectively decrease tumor growth in animal experiments conducted in a living organism (Carbonetti et al., 2020). EI-05, a FABP5 activator, boosts lipid droplet formation and interferon-β production, thereby enhancing the anti-tumor function of macrophages under inflammatory conditions. Administering EI-05 effectively suppresses the development of breast cancer cells (Rao et al., 2015). However, there is vast description of the therapeutic drugs targeting lipid metabolism for the treatment of cancer, so we have summarized these drugs on the basis of targets in Table 1.

**TABLE 1:** Therapeutic drugs targeting key molecules of altered lipid metabolism in different types of carcinoma.

Drug	Lipid metabolism target	Cancer	Study type	Cancer model	Reference
TVB-2640	FASN	Lung, ovarian and breast cancer	Clinical (NCT02223247)	Human samples	Falchook et al. (2021)
		Glioblastoma	Clinical (NCT03032484)	Human samples	Kelly et al. (2023)
C75		Colon cancer	Preclinical	CACO-2, LOVO, HT-29, and HCT-116 cells; tissue samples	Accioly et al. (2008)
		Medulloblastoma	Preclinical	NeuroD2-SmoA1 mice bearing tumors; CGNP cultures	Bhatia et al. (2011)
		Breast cancer	Preclinical	4T1, EMT6, LLC1, B16F10, KLN-205, A20, and CT26 cells; Female Balb/c, Balb/c nude, C57BL/6, and DBA/2 mice	Sun et al. (2021)
		Gastrointestinal stromal tumors	Preclinical	GIST48, GIST430 cells; tissue samples; SCID mice	Li et al. (2017a)
		Thyroid cancer	Preclinical	TPC-1 and FTC-133 cells	Rabold et al. (2020)
Cerulenin		Colon cancer	Preclinical	HCT116 and RKO cells; SCID mice	Shiragami et al. (2013)
Orlistat		Pancreatic cancer	Preclinical	PANC-1, AsPC-1, HPAF-II, Capan-1, Capan-2, CFPAC-1, MIA PaCa-2, T3M4, BxPC-3, CFPAC-1, HuPT3, COLO 357, PaTu 8902, SW 1990, and AsPC-1 cells; PANC-1 cells transfected nude mice	Tadros et al. (2017)
		Prostate cancer	Preclinical	LNCaP and PC3 cells; LNCaP or PC3 cells transfected nude mice	Chuang et al. (2019)
		Ovarian cancer	Preclinical	A2780cis cells; A2780cis cells transfected nude mice	Papaevangelou et al. (2018)
		HCC	Preclinical	Huh7, SMMC-7721, and 293T cells; human tissue samples	Li et al. (2023b), Shueng et al. (2022)
		NSCLC	Preclinical	PC-9, HCC4006, H1650, H820, and H1975 cells; PC-9GR and H1975 NSCLC cells transfected NOD scid gamma (NSG) mice	Ali et al. (2018)
TVB-3664		HCC	Preclinical	Nude mice	Wang et al. (2022)
		Lung adenocarcinoma	Preclinical	Human tissue samples	Liu et al. (2021)
Betulin	SREBPs	HCC	Preclinical	HepG2, Hu7, SMMC-7721, and BEL-7402, MHCC97 cells; HCC cells transfected nude mice	Li et al. (2017b), Yin et al. (2019)
Silibinin		Prostate cancer	Preclinical	LNCaP, DU145, and PC3 cells	Nambiar et al. (2014)
		Endometrial carcinoma	Preclinical	Ishikawa and RL-952 cells; RL-952 cells transfected nude mice	Shi et al. (2019)
Fatostatin		Prostate cancer	Preclinical	LNCaP, PC-3, DU145 and C4-2B cells; Nude mice bearing prostate cancer cells	Li et al. (2014), Li et al. (2015)
		Breast cancer	Preclinical	MCF-7, T47D, BT20, MDA-MB-231 cells; MCF-7 cell xenografts	Brovkovych et al. (2018)
		Melanoma	Preclinical	A375, M202, M207, M233, 451, M229, M238, and M249 cells; Mel006 PDX model	Talebi et al. (2018)
Soraphen A	ACC	Prostate cancer	Preclinical	LNCaP and BPH-1 cells	Beckers et al. (2007)
ND-646		NSCLC	Preclinical	A549, H157, H1355, A427, H23, H460, H358 and H1299 cells; A549 tumor xenograft	Svensson et al. (2016)
TOFA		Head and neck squamous cell carcinoma (HNSCC)	Preclinical	HN5, FaDu, Tu159, OSC19, MDA 1986, UMSCC1, and Tu167 cells; human tumor specimens	Luo et al. (2017)
		Lung and colon cancer	Preclinical	NCI-H460, HCT-8 and HCT-15 cells	Wang et al. (2009a)
		Prostate cancer	Preclinical	LNCaP, C4-2b, CWR22Rv1, PC3, DU145 cells	Guseva et al. (2011)
SB-204990	ACLY	Ovarian cancer	Preclinical	A2780, SKOV3, and HEY cells; A2780 and A2780/CDDP cells xenograft	Wei et al. (2021)
		Melanoma	Preclinical	WM35, WM793B, 451LU, A2058, A375, UACC62, UACC257 cells; A2058 or 451Lu transfected nude mice	Guo et al. (2020)
Simvastatin	HMGCR	Prostate cancer	Preclinical	LNCaP cells; Tumor xenograft	Zhuang et al. (2005)
		NSCLC	Clinical (NCT00452244)	Human samples	Han et al. (2011)
		Breast cancer	Clinical (NCT04418089)	Human samples	Yulian and Siregar (2021)
		Prostate cancer	Clinical (NCT04026230)	Human samples	Siltari et al. (2022)
		Endometrial cancer	Preclinical	RL95-2, HEC1B, and Ishikawa cells	Kim et al. (2019)
		RCC	Preclinical	786-O, SN12C and A498 cells; 786-O cells transfected SCID mice	Hagiwara et al. (2018)
		Glioblastoma	Preclinical	U87 and U251 cells	Shojaei et al. (2020)
Lovastatin		Gallbladder cancer	Preclinical	GBC tumor tissues; NOZ cells transfected nude mice	Zhang et al. (2019)
		Nasopharyngeal carcinoma	Preclinical	5–8F and 6–10B cells	Peng et al. (2017)
dmrFABP5	FABP5	Prostate cancer	Preclinical	DU145, PC3 and LNCaP cells; PC3-M cells were transfected nude mice	Al-Jameel et al. (2019)
SBFI-102 and SBFI-103		Prostate cancer	Preclinical	PC3, DU-145, and 22Rv1 cell-lines; prostate cancer cells transfected BALB/c nude mice	Carbonetti et al. (2020)
EI-05		Breast cancer	Preclinical	E0771 cell line transfected C57BL/6 mice	Rao et al. (2015)
BMS309403	FABP4	Ovarian cancer	Preclinical	SKOV3ip1, HeyA8, 293T, OVCAR5, OVCAR8, and CAOV3cells; OVCAR8 transfected C57/Bl6 mice	Mukherjee et al. (2020)
ABT-510	CD36	Glioblastoma, melanoma, RCC	Clinical (NCT02573324, NCT00073125)	Human samples	Nabors et al. (2010), Ebbinghaus et al. (2007)
SSO		Leukemia	Preclinical	CML Mouse Model	Ye et al. (2016)
A939572	SCD	RCC, Lung cancer	Preclinical	RWV366T and KIJ265T; A498 cells transfected nude mice; A549, H838 and H1573 cells; A549 cells transfected nude mice	von Roemeling et al. (2013), She et al. (2019)
		Glioblastoma	Preclinical	T98G-R, U87-R, T98G, U87, U251, U343, MGR2, and Hs683 cells	Dai et al. (2018)
g-PPT		NSCLC	Preclinical	Human tissue samples; HCC827, PC-9, and H1975 cells	Huang et al. (2019)
Thiazolidinedione	PPARγ	Colon cancer	Preclinical	HT-29 cells	Shimada et al. (2002)
		Pancreatic cancer	Preclinical	AsPC-1, BxPC-3, Capan-2, HPAF-II cells, Capan-1, AsPC-1, PANC-1, and MIA PaCa-2 cells	Eibl et al. (2001), Elnemr et al. (2000)
GW9662		Bladder cancer	Preclinical	Human tissue samples: 5637, T24, UM-UC-3 cells	Cheng et al. (2019)
Nelfinavir	S1P/S2P	Liposarcoma	Preclinical	SW872, LiSa-2 cells; LiSa-2 cells transfected SCID mice	Guan et al. (2011)
Nelfinavir		Prostate cancer	Preclinical	DU145 and PC-3 cells	Guan et al. (2015)

## Conclusion

It is widely accepted that altered lipid metabolism plays a role in cancer metabolism. We explored significant and developing processes in lipid metabolism that promote cancer growth and advancement, as well as prospective targets of oncogenic signaling pathways. Here we have summarized the potential therapeutic strategies targeting the crosstalk between oncogenic signaling and lipid metabolism to inhibit cancer progression and metastasis. Dysregulated lipid metabolism plays a crucial role in tumor metabolic adaptability and contributes to therapeutic resistance, metastatic dissemination, and secondary tumor growth. The local TME is crucial because lipids from nearby tissues play a significant role in both primary and secondary tumor formation, emphasizing the importance of lipids in cancer progression. Tumors experience a change in lipid metabolism, which is closely associated with the development of malignancy and the advancement of the tumor. Lipid metabolic reprogramming is closely linked to oncogenic signaling in cancer cells. The activation of oncogenic signals can induce the expression of genes linked to lipid metabolism, which subsequently controls lipid metabolism in cancer cells. Lipid metabolic by-products can increase the activation of cancer-causing pathways, leading to increased cancer cell growth, invasion, and spread to distant parts of the body. There have been significant advancements in the identification of therapeutic targets for lipid metabolism in cancer therapy. The interaction between lipid metabolism and important signaling mediators in the environment of cancer is a highly interesting topic of study. An in-depth understanding of how dysregulated lipid metabolism influences cancer progression is crucial for the development of innovative cancer therapy strategies. It is clear that focusing on a single component or pathway of lipid metabolism is unlikely to lead to more effective cancer treatment. Developing treatments that can impede tumor growth and boost the body’s ability to fight tumors is a highly promising strategy for lipid-based medicinal research. Little information is available regarding how oncogenic signaling pathways indirectly influence the cancer microenvironment or the lipid metabolism it activates. Investigating the relationship between modifying medicines and lipid metabolism will be a promising area of future cancer research.
